# Feasibility of the adaptive and automatic presentation of tasks (ADAPT) system for rehabilitation of upper extremity function post-stroke

**DOI:** 10.1186/1743-0003-8-42

**Published:** 2011-08-03

**Authors:** Younggeun Choi, James Gordon, Hyeshin Park, Nicolas Schweighofer

**Affiliations:** 1 Division of Biokinesiology and Physical Therapy, University of Southern California, Los Angeles, California, USA; 2 Department of Computer Engineering, Dankook University, Yongin, Gyeonggi-do, South Korea

## Abstract

**Background:**

Current guidelines for rehabilitation of arm and hand function after stroke recommend that motor training focus on realistic tasks that require reaching and manipulation and engage the patient intensively, actively, and adaptively. Here, we investigated the feasibility of a novel robotic task-practice system, ADAPT, designed in accordance with such guidelines. At each trial, ADAPT selects a functional task according to a training schedule and with difficulty based on previous performance. Once the task is selected, the robot picks up and presents the corresponding tool, simulates the dynamics of the tasks, and the patient interacts with the tool to perform the task.

**Methods:**

Five participants with chronic stroke with mild to moderate impairments (> 9 months post-stroke; Fugl-Meyer arm score 49.2 ± 5.6) practiced four functional tasks (selected out of six in a pre-test) with ADAPT for about one and half hour and 144 trials in a pseudo-random schedule of 3-trial blocks per task.

**Results:**

No adverse events occurred and ADAPT successfully presented the six functional tasks without human intervention for a total of 900 trials. Qualitative analysis of trajectories showed that ADAPT simulated the desired task dynamics adequately, and participants reported good, although not excellent, task fidelity. During training, the adaptive difficulty algorithm progressively increased task difficulty leading towards an optimal challenge point based on performance; difficulty was then continuously adjusted to keep performance around the challenge point. Furthermore, the time to complete all trained tasks decreased significantly from pretest to one-hour post-test. Finally, post-training questionnaires demonstrated positive patient acceptance of ADAPT.

**Conclusions:**

ADAPT successfully provided adaptive progressive training for multiple functional tasks based on participant's performance. Our encouraging results establish the feasibility of ADAPT; its efficacy will next be tested in a clinical trial.

## Background

Over 80% of first-time strokes (infarctions only) involve acute hemi-paresis of the upper limb [1]. There is now definite evidence that intensive task-specific practice is effective in improving upper extremity function and use after stroke [2]. In current clinical settings, however, at least in the US, patients with stroke receive only a small fraction of the dose of therapy needed for effective rehabilitation of upper extremity functions [3-6]. In view of this shortcoming of the conventional medical practice model, a growing number of robotic systems for the rehabilitation of upper extremities after stroke are being developed and tested [7-17]. Rehabilitation robots can potentially provide an automated and cost-effective addition to human therapists to increase the duration and intensity of therapy in rehabilitation settings.

Here, we argue that to further enhance the efficacy of therapy targeting the recovery of upper extremity functions, the design of rehabilitation robotic systems could benefit from three evidence-based rehabilitation principles: 1) training should maximize active participation from the patient; 2) training should involve practice of real-world tasks; and 3) training should be individualized by adaptively changing the number of trials and difficulty of each task practiced according to the evolving skill level of the patient.

### Active participation training

The beneficial effects of self-produced movements over passive movements in the development of visually guided behavior have been long known [18]. More recent work has shown that motor cortex reorganization underlies motor improvements during active motor training [19-22]. Although passive movements also elicit activity in the motor cortex, e.g. [23], actively generated movements are more effective in eliciting both performance improvements and cortical reorganization [24]. Along these lines, training with active participation and repetitive practice has been shown to be effective in stroke rehabilitation [25]. Recognizing that pure assistance may not be entirely beneficial for motor learning and recovery of arm and hand function [26,27], rehabilitation roboticists have started to balance assistance provided by the robot with active movement by the patient, e.g. [28-30].

### Task oriented training

Recovery of functional tasks constituting activities of daily life has been shown to be critical for patients in their attempts to return to a reasonable quality of life [31,32] and improved participation [33]. It is well established that the best way to learn an activity is to practice that activity, which means task-specific training [34], and there is mounting evidence that functional and meaningful task training is effective in stroke rehabilitation [35-38] (but see [39]). In particular, the manipulation of real and functional objects seems to be beneficial [40,41]. As a result, Task-Oriented Training (TOT) has emerged as the dominant approach to motor restoration (NINDS Stroke Progress Review Group, 2006). To our knowledge, only two automated upper extremity rehabilitation systems allow functional task training with manipulation of real objects. First, AutoCite, which is a semi-automated (non robotic) system that allows patients to engage in the practice of functional tasks, has been shown to be as effective as standard constraint-induced (CI) therapy [42,43]. AutoCite's hardware and software is capable of automatic task selection and difficulty adjustment based on previous performance patterns. However, in the preliminary testing with patients, no adaptive algorithm was tested, and task selection and adjustment of difficulty was done manually. Second, the robotic system ADLER presents multiple functional tasks for activities of daily living [44]. Note however that ADLER requires a therapist to set up the different objects to be manipulated.

### Individualized training

In motor learning, retention of skill over a prolonged period is strongly influenced by the number of trials and by the schedules in which the multiple tasks are practiced (e.g., random, blocked or interval-expanded presentation of tasks) [45-47]. Furthermore, challenging tasks, that is, tasks that are neither too difficult nor too easy, are most likely to elicit motor learning [19,48-50]. Challenging tasks also enhance motivation, which may in turn further enhance learning [51]. Because performance typically improves during skill acquisition according to negatively accelerated learning curves (but not always, see for instance [52]), and because learning evolves at different rates for each task and each subject, task difficulty needs to be dynamically adjusted to maintain challenge at an optimal level. During neuro-rehabilitation, it is likely that physical and occupational therapists adaptively modify task practice parameters using intuitive and largely implicit rules [53]. Along these lines, a number of adaptive difficulty algorithms based on performance have been implemented on robotic systems [28,54,55].

In previous work, we developed a novel robotic system, the ADaptive and Automatic Presentation of Tasks (ADAPT) system, which implements all three rehabilitation principles mentioned above: ADAPT allows active and individualized training on a number of real-world functional tasks [56]. ADAPT can accommodate an expanding number of such tasks, and it allows the implementation of performance-based adaptive task scheduling and task difficulty by simulating the dynamics of daily living functional tasks, such as opening a doorknob, opening a jar, turning a key, etc, each of which requires active reaching and manipulation.

The different tasks and tools were designed to engage training of different grasps (power, overhand, and pinch) and arm movements. Thus, like other automated rehabilitation devices, ADAPT allows both delivery of large intensity of training and longitudinal measurements of performance and limb use. However, the design of ADAPT departs from that of most other current robotic systems. In these systems, the task goal is generally pre-specified, and the robot modulates the assistance provided to the patient to attain that goal. Instead, ADAPT provides assistance-free training modeled after TOT. In this form of training, the rehabilitation therapist selects salient tasks for the patient to work on, adjusts the task parameters by increasing task difficulty and complexity as learning progresses, and provides informative and motivational feedback. Accordingly, in ADAPT, the task difficulty is constantly adapted such that patient always actively performs doable, but challenging, tasks.

The primary aim of this feasibility study was to establish the feasibility of ADAPT for a single training session of participants with chronic stroke. Specifically, we evaluated ADAPT's safety, ADAPT's overall functionality, possible improvement of performance between pre- and post-test, and participants' subjective experience.

• Safety was measured both quantitatively by the number of adverse event occurring in the operation of the ADAPT and qualitatively via a participant questionnaire similar to that used in other robot acceptance studies such as in [57,58].

• Functionality, which is broadly defined as the robot applicability toward the accomplishment of a task [59], was evaluated in three ways. First, we tested whether ADAPT could successfully present the different tasks to the participants without human intervention. Second, we evaluated the fidelity of the dynamics of the simulated tasks both by comparing it to actual task dynamics and via questionnaire. Third, we evaluated whether the adaptive algorithm could successfully modulate task difficulty based on performance during training.

• Improvement in performance was measured by the time it takes the subject to perform the 6 different tasks between pre- and post-test.

• Participants' subjective experience was assessed via the Intrinsic Motivation Inventory (IMI) questionnaire, [51,60,61] and via the participant questionnaire.

We limited our target population to individuals with chronic stroke who have some residual arm and hand movements; that is, participants in our study can be classified as "moderately to mildly" impaired [2]. Such participants were included for two reasons. First, because the safety of ADAPT had only been tested with healthy participants, we included participants who had the potential to easily disengage themselves from the task whether an unexpected robot behavior would occur. Second, because ADAPT does not provide any physical assistance, but instead require active engagement to perform reach, grasp, and manipulate objects, it is designed for patients with residual volitional motor capability of the arm and hand.

## Methods

### Participants

Five participants (age 66.2 ± 3.3 years, one female) with chronic stroke (time since stroke 6.3 ± 2.3 years) signed an informed consent to participate in this study, which was approved by the IRB at the University of Southern California. The inclusion criteria were: a single episode of stroke at least 9 months prior; 90 degree of shoulder flexion and 30 degree of elbow extension. The exclusion criteria were: serious uncontrolled medical conditions; excessive pain in any joint of the more affected extremity that could influence participation in the tasks; a score of less than 25 on the Folstein Mini Mental State Examination; demonstration of a less than thorough understanding of the instructions; Fugl Meyer (FM) arm score < 40; impossibility to perform at least four tasks at their lowest difficulty in the pre-test with ADAPT (see below). The average Fugl Meyer arm score was 49.2 ± 5.6 (SD), in the range 42 to 56, extension (see Table 1 for summary of patient characteristics).

**Table 1:** Participants characteristics. Hand dominance is before stroke

Subject ID.	Age (years)	Gender	Months post-stroke	Fugl-meyer score (0-66)	Affected hand	Hand dominance
S1	54	M	46	40	Center	Right

S2	77	M	63	56	Center	Right

S3	63	M	83	51	Right	Right

S4	65	M	54	45	Right	Right

S6	68	M	78	42	Center	Center

### The ADAPT robotic system

**Figure 1:**
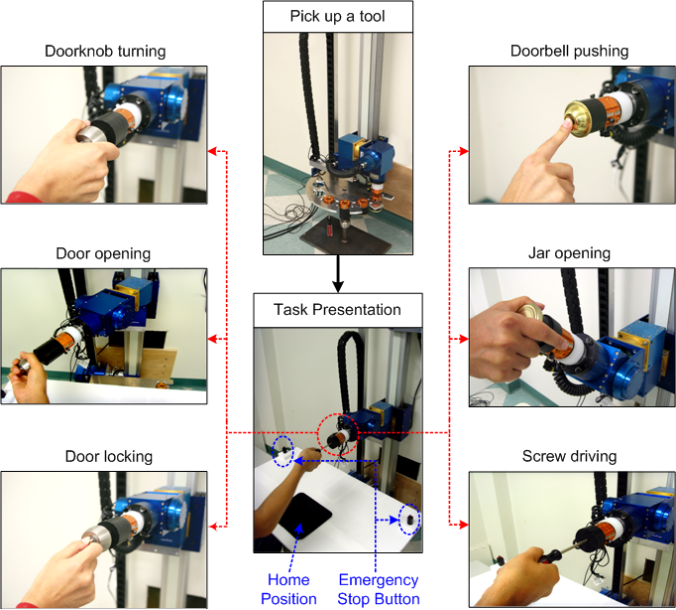
**Current implementation of the tool-changing process and functional task in ADAPT**. Six reach-to-grasp tasks with four different functional task tools were implemented (doorbell: bell pushing; jar: jar opening; doorknob: knob turning, door locking, door opening; screwdriver: screw driving).

Here, we only briefly describe ADAPT and its adaptive difficulty scheduling capabilities, since we previously reported details of ADAPT's design and control systems [56]. In its current configuration, ADAPT is a general-purpose robot (Amtec Robotics) with a 3-DOF wrist mounted on a 1-DOF linear actuator. Note that, unlike many rehabilitation robots, this robot has low backdrivability; this allows the robot to generate high torques and makes it easier for the robot to automatically pick up new tools, at the price of reduced haptic fidelity, however. The control architecture of ADAPT contains three modules. The *high-level adaptive task scheduler *of ADAPT selects both the task to practice within the task bank and the task difficulty based on the patient's previous performance. Four task tools (door knob, doorbell, jar, screw driver) are used to implement six tasks (listed in Table 2). The tools are arranged in a rack from which the robot picks up a tool for a selected functional task (Figure 1). Once the task is selected, the scheduler sends a command to the tool-changing system that picks up the tool corresponding to the selected task. The tool changer is locked or unlocked by a pneumatic valve to switch between the current tool and the next tool in the rack.

**Table 2:** List of Functional Tasks

*Task*	*Description*	*Performance metric*	*Tool*
**Doorknob turning**	Turn a door knob with power grasping to the end of turning range, and release it.	Angle	Knob

**Doorbell pushing**	Push a door bell with a finger over a threshold force.	Force	Doorbell

**Jar opening**	Turn a jar with power grasping up to the end of turning range.	Angle	Jar

**Door opening**	Move a door knob horizontally with power grasping. Mostly elbow and shoulder motion.	Angle	Knob

**Door locking**	Turn a door knob button with pinch grasping to the end of turning range.	Angle	Knob

**Screwdriving**	Turn a screwdriver with power grasping to the end of turning range.	Angle	Screw driver

The *functional task model *generates a desired trajectory to simulate the dynamics of the selected task with the difficulty specified by the high-level adaptive task scheduler. The dynamics of a functional task is expressed as:(1)

where *τ *is the torque exerted by a subject, *Diff *is an adjustable difficulty variable, *q*, ,  are angle, velocity, acceleration respectively of task motion, and  is the dynamics model of an original functional task. For three tasks (doorknob turning, jar opening and door-locking tasks), we modeled the functional task dynamics using a constructive locally weighted method, such that non-linear dynamics (like friction) are accurately modeled to simulate the feel of task tools [56]. This approach provides computationally efficient way of modeling non-linear dynamics via the combination of multiple local linear models by adaptively creating and pruning the local models (see [56] for details). For bell pushing and door opening, we used simple mathematical models;for screw driving we reused the jar opening model captured by our original model by changing the parameters of the local linear models empirically.

At each trial, the high-level adaptive task scheduler adaptively changes the task difficulty by controlling the *Diff *in equation (1). The update function for the variable *Diff *in equation (1) is given by(2)

where *Difft, k *is the difficulty for the current trial *t *and the task *k, α *is learning rate, *Perft-1, k *is the performance on the previous trial, and *Perfref, k *is the reference challenging performance. Task difficulty is updated to constantly maintain performance around the "challenging point", *Perfref, k *(see [45] for a detail of this algorithm).

The *low-level admittance controller *computes the control current corresponding to the desired trajectory to implement the desired dynamics of the task with difficulty prescribed by the high-level controller. The computed control current is then applied to the robot to simulate the task, and the patient feels the simulated dynamics by manipulating the selected tool (see [56] for details).

Safety was a crucial issue in the design of ADAPT. Our choice of design makes our robot safer than a traditional multi-DOF robotic arm because of the small overall workspace. The linear DOF is only used for tool positioning, not for task dynamics simulation. For simplicity and safety reasons, we choose tasks that require movements around a single DOF during subject-robot interactions. After a task is set up for presentation to the subject, the magnetic brakes that are built into the robotic articulations are engaged on the other three DOFs during subject-robot interactions. Furthermore, the patient is not strapped to the robot but freely interact with the robot, only after the robot positions the tool.

Several surveillance routines are implemented to limit the maximal torque output and cap the maximum velocity of the linear and rotational motors. Watchdog routines that continuously check for failure of the position and force sensors, computer crashes, and electrical failures automatically freeze the robot by engaging the magnetic breaks in all DOFs. When pressed, two emergency stop buttons stop all robot operation and turn on magnetic brakes to disable any movement of all 4 DOF of the robot. The main emergency red stop button of the power box in is accessible to the therapist. The subject holds the second emergency stop button at all times with their less affected hand.

### Experimental Procedures

Participants sat on a chair in front of ADAPT and were instructed, at each trial, to reach and manipulate a functional task tool, which was selected by the adaptive scheduler and then picked up and presented by the robot. The experiment started with a pre-test followed by a training session, and ended with a post-test after one hour break. In the pre-test and post-test, participants practiced all six tasks without feedback with difficulty set at the easiest levels, in block of three trials each (total 18 trials). Then, four tasks were selected from the six tasks of the pre-test for the training session. If a participant could not complete a specific task in the pre-test, that task was not selected. Otherwise, the four tasks were selected pseudo-randomly, so as to counterbalance the number of tasks across participants. The training session, which lasted about one and half hour, consisted of 48 blocks of three trials (12 blocks per task), pseudo-randomly scheduled so that each task occurred once in any four consecutive blocks.

**Figure 2:**
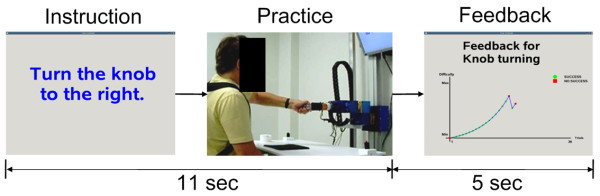
**Experiment procedure with ADAPT**. After visual and audio instructions, the participants reached and manipulated the selected tool. Note that feedback showing the difficulty and success for each task was displayed only after blocks of three trials.

At the beginning of each trial, we instructed the participants to place their affected hand on the home position (Figure 1). Then, a combined visual and auditory instruction was displayed to indicate the task to be practiced. The participants were then instructed to reach and manipulated the selected tool at self-selected speed (see Figure 2). We did not control for the participants strategy in this feasibility study, but all movements were video recorded for further analysis of possible compensatory movements. Eleven seconds were allowed for the completion of one trial, at the end of which the participants received an auditory feedback signal indicating success or failure. After every block of three trials for each task, feedback of the participants' progress for all practiced tasks was displayed (Figure 2). The next trial started after the participants' hand was back on the home position.

Fidelity is the extent to which the appearance and behavior of the simulator/simulation matches the appearance and behavior of the simulated system. We tested the two components of fidelity: engineering fidelity, which is 'the degree to which the simulator replicates the physical characteristics of the real task', and psychological or functional fidelity, which is 'the degree to which the skill or skills in the real task are captured in the simulated task'[62]. We studied engineering fidelity by checking whether the tasks have the qualitative mechanical characteristics of the tasks modeled. We quantified psychological fidelity via the participant questionnaire.

The participants practiced the selected four tasks in the adaptive difficulty mode, in which the difficulty of each task was updated according to equation 1. Gradual and progressive motor adaptation promotes longer-lasting effect in motor learning compare to sudden adaptation [63]. Therefore, the task difficulty was set to be lowest initially, and increased progressively based on performance. Additionally, this set up led to high probability of success in initial trials, which is presumably important for high motivation [51]. Due to a high learning rate in equation (1), task difficulty increased rapidly, so that performance reached the challenging point before the end of training in most cases. We recorded the number of trials need for the difficulty level to stop increasing and then, for the remaining trials, the deviation from the challenge point.

We assessed improvements in performance due to training by comparing median movement times (over three trials) on the tasks in the pre- and post-tests. Because all six tasks were given in the pre- and post-tests, but only four tasks were practiced during training, there were 20 out of 30 (five participants by six tasks) trained tasks, and 10 untrained tasks. We performed paired sample t-tests on the median movement times to analyze the effect of training. We report results as means ± standard deviations. Our significance level was p < .05.

Following the post-test, two questionnaires were administered to evaluate the acceptance of ADAPT. We first administered the Intrinsic Motivation Inventory (IMI) questionnaire, which was designed to assess participants' subjective experience related to a target activity in laboratory experiments [51,60], and has been previously used to measure stroke patients' experience in robotic training [61]. Second, we administered a specific questionnaire, similar to those used in other robot acceptance studies such as in [57,58], to inquire about perceived safety, fidelity of simulated tasks and subjective experience.

## Results

### Safety

All five participants completed the robotic training sessions, functional measurements, and questionnaires. Safety concerns were strongly addressed from the initial design process of ADAPT (see above and [56]), and no adverse event occurred during direct interaction between participants and ADAPT. In particular, the two safety buttons were never activated. Finally patient reported very high sense of safety in the post-training questionnaire (6.80 ± 0.45 out of 7).

### Automatic presentations of functional tasks without human intervention

ADAPT could successfully present the six tasks to all 5 participants without human intervention. The robot thus implemented a total of 900 trials (18 pre-test trials + 48 * 3 training trials + 18 post-test trials for 5 subjects) without any failure either in the robot itself or in the pneumatic tool changer.

### Fidelity of Functional Task

**Figure 3:**
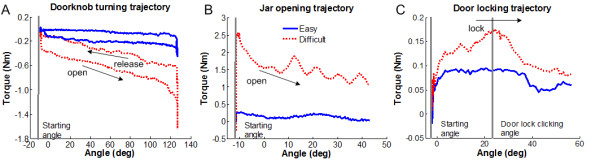
**Sample trajectories of functional tasks**. Torque versus position trajectories are plotted for three functional tasks in the training session. The blue solid line shows the trajectory of each task at lowest difficulty, which is selected in the first trial of the training session. The red dot line is the trajectory of each task at high difficulty, which is selected near the end of the training session.

The engineering task fidelity was qualitatively demonstrated by showing that the actual tasks had the characteristics of the simulated tasks for variable task difficulty. We plotted the torque versus angle trajectories for three representative tasks (door locking, doorknob turning, and jar opening) in Figure 3. The sample trajectories at two different levels of task difficulty were selected from trials in the training session of one participant (FM score: 45). The door locking task followed a spring-like torque, which increased proportionally to rotated angle from the initial point until it passed the lock clicking point (Figure 3a). The doorknob turning task followed a typical spring whose torque was proportional to the rotated angle (Figure 3b). The jar opening task followed a static friction torque, which served as an initial large threshold, and a small dynamic friction torque during rotation (Figure 3c). The screw driving task followed a friction dynamics similar to jar opening task. The doorbell pushing task was implemented by playing a bell sound when the pushing force reached a specific threshold in limited time. Finally, the door opening task followed a pure damping and used the second DOF of the robot to elicit subject's arm movements from left to right in the horizontal plane.

Participants reported that they could clearly understand how to interact with ADAPT through auditory and visual instructions, and that the simulated functional tasks were fairly similar to real tasks (participants scored 5.90 ± 1.6 out of 7 when asked if the tasks were similar to real functional tasks). One participant verbally reported that pushing bell required too much force, suggesting that tasks with high difficulty could be unrealistic.

### Maintaining challenging performance via adaptive difficulty

**Figure 4:**
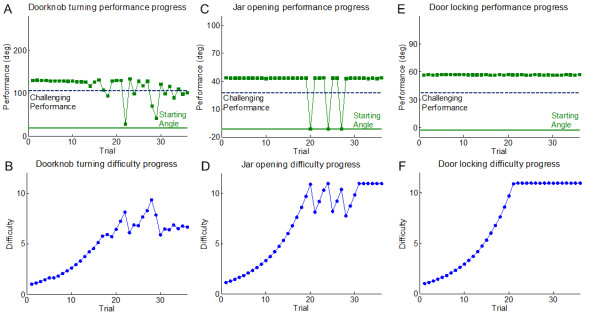
**Progress of Performance & Difficulty**. Illustration of progress in performance and difficulty as a function of trials for a representative participant (FM score: 45) during the training session. Initial difficulty was initially set at its lowest value, and increased adaptively depending on performance. In the doorknob turning task (A), performance was maintained near the challenging point from around 15 trials. In the jar opening task (B), the participant failed to complete the task for three trials near the highest difficulty, but could then complete the task near the end of training. The door locking task (C) was too easy for the participant at all difficulty settings.

The adaptive difficulty schedule in the training session aimed to maintain participants' performance near a challenging performance point. Figure 4 shows examples of performance and adaptive difficulty in the training session for one participant (FM score: 45) for doorknob turning, jar opening, and door locking, respectively. In Figure 4a, the algorithm progressively increased the task difficulty based on initial successful performance, and adaptively responded to participant's performance by increasing or decreasing the difficulty by the right amount of change to maintain the performance near the challenging point. In Figure 4b, the algorithm also adaptively modulated the difficulty based on performance. The participant started to fail to complete the task near maximum difficulty. Following several trials of failure and success with modulation of the difficulty, the participant succeeded in later trials. In Figure 4c, the participant succeeded in all 36 trials of the door locking task.

We then analyzed the performance of the adaptive algorithms for each task across participants. Since the difficulty level was set at the easiest level for all tasks and participants, it took an average of 29.4 ± 8.2 trials for the difficulty level to stop increasing. Then for the remaining trials, difficulty stayed within 33.3 ± 27.9% of the challenge point.

### Improvements in movement time

**Figure 5:**
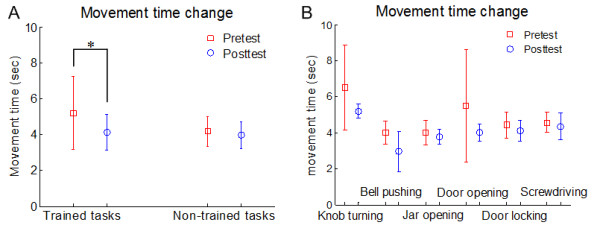
**Movement times in pre- and post-test sessions**. In (A), we compared movement times between pre-test and post-test to measure the effect of training session. In (B), we compared movement time between pre-test and post-test for each task.

Movement times decreased from the pre- to post-test in average for all trained tasks (pre: median 5.18 ± 2.04 sec to post: 4.12 ± 1.00 sec, *p *= 0.022), but not for non-trained tasks (4.17 ± 0.84 sec, 3.96 ± 0.74 sec, *p *= 0.105) (Figure 5). For all six tasks, the mean movement time appeared to decrease from pre- to post-test, although the small number of samples prevents the computation of significance values for individual tasks (Figure 5).

### Acceptance of ADAPT

Table 3 shows the mean scores on the IMI questionnaire. The high score of the interest/enjoyment of IMI implies that our participants found the training session with ADAPT interesting. The high score of effort/importance and value/usefulness subscales indicates that participants were highly motivated and satisfied with the experience and the result from the training with ADAPT. The low score of pressure/tension subscale means that participants did not feel much pressure or tension during the training with ADAPT. Relatively low score of the perceived/competence may be due to the different levels of disability of our participants, in line with the results from a previous study [61].

**Table 3:** Subscale findings of the IMI questionnaire administered after training (subscale range = 1 - 7)

*Subscale*	*Score (Mean ± SD)*
**Interest/Enjoyment**	6.17 ± 1.25

**Perceived Competence**	4.90 ± 1.88

**Effort/Importance**	6.32 ± 0.75

**Value/Usefulness**	6.34 ± 1.43

**Pressure/Tension**	1.84 ± 0.94

**Perceived Choice**	6.43 ± 1.40

Results from the second questionnaire also suggest, in general, that training with ADAPT was well accepted and well tolerated by our participants (Table 4). While there were a few complaints about the seatbelt, participants mostly felt comfortable interacting with ADAPT.

**Table 4:** Patients' acceptance for the training session with ADAPT (subscale range = 1 - 7)

*Question*	*Score (Mean ± SD)*
**Comfortable with robot's sound & appearance**	6.90 ± 0.32

**Safety**	6.80 ± 0.45

**Comfortable with seatbelt & chair**	6.10 ± 1.52

**Similar to real functional tasks**	5.90 ± 1.62

**Fatigue or Frustration**	3.40 ± 1.95

**Clear instructions**	7.00 ± 0.00

## Discussion

In this study, we tested and evaluated the feasibility of ADAPT with five participants with chronic stroke during one day training session. Specifically, we evaluated safety, overall functionality, fidelity of simulated tasks, improvement in performance, and patient acceptance.

Safety was excellent since no adverse event occurred during training, and the emergency stop buttons were needed by neither the participants nor the experimenter. Results from questionnaire showed excellent perceived safety. Note that the robot exerts large movements when picking up new objects, but such robot movements were programmed at low speed. We also fixed the work table between the robot and the participants such that accidental contact between the robot and the participant during this phase is unlikely.

For all participants and all tasks studied, ADAPT could accomplish the following functions satisfactorily. 1. Present a variety of functional tasks without human intervention 2. Simulate the dynamics of these functional tasks and 3. Modulate adaptive task difficulty based on performance during training,

First ADAPT could present a variety of functional tasks without human intervention for a total of 900 trials. This shows the feasibility of our novel tool changing system, based on a pneumatic tool changer, which automatically selected one of four tools based on the task schedule. No failure with the tool changing process occurred for all five participants' testing and training sessions. Extension to a greater number of tools in future work is straightforward.

Second, ADAPT could simulate the dynamics of the functional tasks qualitatively, as demonstrated by showing that the actual tasks had the characteristics of the simulated tasks for all six tasks. In addition, participants reported good, although not excellent, perceived fidelity. Two reasons may have led to this result. First, we modeled the functional task dynamics with constructive locally weighted method only for doorknob turning, jar opening and door-locking. In contrast, we used simple mathematical models for bell pushing and door opening, and we reused the jar opening model for screw driving by changing the parameters of the local linear models empirically. Second, we required large, and increasingly large, amount of force for most tasks. The difficulty of current tasks was mostly proportional to torque or force to be exerted for manipulation. Although strength is beneficial for stroke recovery [37,64], many functional tasks do not require much motor strength, but instead emphasize skill in fine motor coordination. The strength-oriented difficulty algorithm that we used here may cause a task to be perceived as more difficult than the same task in the real world. In future work, we will need to develop tasks that require fine motor skills and derive algorithms that modulate difficulty based on measures of skill of performance such as movement time and/or errors, not strength.

Third, testing of adaptive modulation of task difficulty based on performance during training showed that the adaptive algorithm could reasonably well adapt difficulty to the participant's performance following the initial increase in performance, as performance stayed within 33% of the challenge performance, on average. Such deviation from the challenge performance can occur both as a result of participants behavior (trial-by-trial noise and actual change in performance, due to learning or fatigue) and as a result of the form of our adaptive difficulty updating algorithm (equation 2) and its learning rate parameter *α*, which was set based on data from healthy participants. If the learning rate is too high, the difficulty will follow the noise in the data and large oscillations may occur (possibly as observed in Figure 5b); if too low, the difficulty will lag performance. Additional computer simulations and experiments are needed to define adaptive rules that reduce the deviation between desired and actual performance and capturing actual slow changing changes in participants' performance. Similarly, the parameters for challenging performance *Perf_ref,k _*were also set empirically for each task based on pilot data with healthy subjects. Indeed, how to determine the challenge point parameters systematically is an open question [65]; in addition, a challenging performance parameter that exceeds real-life values may not have much functional relevance. The final limitation of our adaptive difficulty algorithm is that comparison of performance between different tasks is difficult, because the performance value is a physical quantity, such as rotated angle or pushing force. Using success rate as a performance metric is a possible solution to this problem, and efforts are underway to develop statistical models of performance for each task and each subject.

We showed that ADAPT could improve a measure of participants' performance, time to movement time of the task trained, in a single session. Note that while we previously showed that adaptive difficulty can presumably outperform fixed difficulty in motor learning [45], the current study was not designed to specifically test whether the adaptive difficulty schedule was effective. Thus, the gains in movement time may however be unrelated to the adaptive algorithm, as the practice itself could have provided the reduction in movement time. Furthermore, although the current ADAPT system allows to implement adaptive schedule in the number of trials, such as that we previously developed in [45], we implemented here a simple pseudo-random schedule in which the trial numbers were equal for all tasks. Our current adaptive task scheduler requires at least a first "fixed session" to determine the number of trials on subsequent sessions based on performance on delayed retention [45]. Here, because safety with stroke participants was not established before this study, we conducted a single session and thus fixed the number of trials. Furthermore, because the retention test was given 1 hour after practice, our results showing improvement in movement time may be due to short-term practice effects and not true learning. In further work, training for more than one session, and a longer post time measurement will be needed to extrapolate to a long-term benefit for stroke patients. Therefore, in its current form, and because we show no transfer to other tasks (even similar ones), a single session with ADAPT should not be viewed as a comprehensive rehabilitation program, but rather a possible automated module within a rehabilitation program.

Because ADAPT in its current configuration does not constrain the types of reaching and grasping movements performed, patients may use compensatory movements to accomplish the task goal. Videos showed that indeed several patients did not manipulate the task tools as instructed, even though the participants knew the appropriate way of manipulation. In the door locking task for instance, which requires pinch grasping, one participant (FM score: 42) used power grasping to succeed in the trial. Thus, on one hand, compensatory movements could be reduced with tools that constrain hand or finger postures and motion monitoring systems. On the other hand, it has been argued that stroke recovery therapy should not focus solely on the realization of normal movement patterns, but should take some compensatory strategy into account to be more effective [66].

## Conclusions

The results from this study validate the feasibility of ADAPT for rehabilitation of arm and hand function after stroke, and provide justification for continued investigation of clinical efficacy. Furthermore, safe automatic presentation of functional tasks with ADAPT showed the potential to engage effective motor learning in stroke rehabilitation. Motor control and learning principles, such as manipulating the schedule of training, can extend the efficacy of robotic neuro-rehabilitation [67]. This motivates our current and future work in developing hypothesis-driven adaptive schedules to select the appropriate task and difficulty at each trial for optimal stroke rehabilitation.

## Competing interests

An invention disclosure, with intent to obtain a patent in the United States, has been submitted by the Stevens Institute for Innovation of University of Southern California.

## Authors' contributions

YC, JG and NS designed ADAPT. YC and NS developed the study design, and wrote the manuscript. JG aided in the study design and in drafting the manuscript. YC and HP performed the experiment. All authors read and approved the final manuscript.
